# Supplementary material for: Programmed disassembly of a microtubule-based membrane protrusion network coordinates 3D epithelial morphogenesis in Drosophila
Source: EMBO J. 2024 Jan 23;43(4):5. doi: 10.1038/s44318-023-00025-w (PMC10897427; doi:10.1038/s44318-023-00025-w)
Supplement: Supplementary file 1 — Movie EV1 [file 44318_2023_25_MOESM1_ESM.zip › Movie EV1/Movie EV1 legend.docx]

**Movie EV1.** **Details of the IPAN structure. 3-16 seconds**: 3D view of high resolution images of αTubulin:GFP at 13h APF. MT protrusions comprise individual MTs that emanate from the apical surface and bundle further basally. **17-26 seconds**: F-actin-based filopodia surround the MT protrusions and form bridges that connect them. **30-34 seconds**: Schematic of the IPAN at ~13-14 APF (25℃). Green: MT protrusions. Red: F-actin. Blue: plasma membrane. See also Fig. 1.
