# Supplementary material for: Programmed disassembly of a microtubule-based membrane protrusion network coordinates 3D epithelial morphogenesis in Drosophila
Source: EMBO J. 2024 Jan 23;43(4):5. doi: 10.1038/s44318-023-00025-w (PMC10897427; doi:10.1038/s44318-023-00025-w)
Supplement: Supplementary file 2 — Movie EV2 [file 44318_2023_25_MOESM2_ESM.zip › Movie EV2/Movie EV2 legend.docx]

**Movie EV2.** **Time-lapse images of CAAX:mCherry (white) in pupal wing between 13 and 24h APF.** Upper left: interepithelial coronal (XY) views of the IPAN. Upper right: optical cross section (YZ) views. Bottom: optical cross section (XZ) views. Note that wing vein structures become visible after apposition of dorsal and ventral epithelium. **Three seconds**: MT protrusions are found throughout the pupal wing. Regions with few/no MT protrusions are destined to become longitudinal veins. **18 seconds**: Longitudinal veins L2-L5 are clearly visible after apposition of dorsal and ventral epithelium. See also Fig. 2A.
