# Supplementary material for: Programmed disassembly of a microtubule-based membrane protrusion network coordinates 3D epithelial morphogenesis in Drosophila
Source: EMBO J. 2024 Jan 23;43(4):5. doi: 10.1038/s44318-023-00025-w (PMC10897427; doi:10.1038/s44318-023-00025-w)
Supplement: Supplementary file 3 — Movie EV3 [file 44318_2023_25_MOESM3_ESM.zip › Movie EV3/Movie EV3 legend.docx]

**Movie EV3. Time-lapse images of CAAX:mCherry (white) and αTubulin:GFP (green) in pupal wing at 13 APF.** See also Fig. 2B, C.
