# Supplementary material for: Programmed disassembly of a microtubule-based membrane protrusion network coordinates 3D epithelial morphogenesis in Drosophila
Source: EMBO J. 2024 Jan 23;43(4):5. doi: 10.1038/s44318-023-00025-w (PMC10897427; doi:10.1038/s44318-023-00025-w)
Supplement: Supplementary file 4 — Movie EV4 [file 44318_2023_25_MOESM4_ESM.zip › Movie EV4/Movie EV4 legend.docx]

**Movie EV4. Time-lapse images of αTubulin:GFP(green) in pupal wing between 13 and 24h APF.** See also Fig. 2D.
