# Supplementary material for: Programmed disassembly of a microtubule-based membrane protrusion network coordinates 3D epithelial morphogenesis in Drosophila
Source: EMBO J. 2024 Jan 23;43(4):5. doi: 10.1038/s44318-023-00025-w (PMC10897427; doi:10.1038/s44318-023-00025-w)
Supplement: Supplementary file 5 — Movie EV5 [file 44318_2023_25_MOESM5_ESM.zip › Movie EV5/Movie EV5 legend.docx]

**Movie EV5.** **Membrane-bound rCD2:RFP (magenta - dorsal) and mCD8:GFP (green – ventral).** **3-4 seconds**: Representative differentially colored dorsal and ventral MT protrusions. **5-13 seconds**: High-resolution 3D rendering of a subset of differentially colored protrusions. Basal protrusion branches from each epithelium interact with similar branches from the opposed epithelium. **17-28 seconds**: Time-lapse images showing IPAN dynamics and apposition of dorsal and ventral epithelia between 13.5h and 25h APF (25℃). Optical cross-sectional view at two different positions. **28 seconds**: The lacunae between the epithelia are cross-sections of longitudinal veins. See also Fig. 3.
