# Supplementary material for: Programmed disassembly of a microtubule-based membrane protrusion network coordinates 3D epithelial morphogenesis in Drosophila
Source: EMBO J. 2024 Jan 23;43(4):5. doi: 10.1038/s44318-023-00025-w (PMC10897427; doi:10.1038/s44318-023-00025-w)
Supplement: Supplementary file 6 — Movie EV6 [file 44318_2023_25_MOESM6_ESM.zip › Movie EV6/Movie EV6 legend.docx]

**Movie EV6. Four stages in cells in which MT protrusions regress, with subsequent mitosis. 4-22 seconds**: Images and 3D renderings of the four stages of MT protrusion regression with subsequent mitosis. White chevrons point at events occurring as stages progress. **27-56 seconds:** Basal view of αTubulin:GFP and Cnn:RFP of time-lapse images of MT protrusions and subsequent mitosis. MT protrusions (27 seconds). Regression of protrusion starts (31 seconds). Cell rounds up (37 seconds). Protrusion has completely regressed (41 seconds). Mitotic spindle forms (46 seconds). Cell in metaphase (49 seconds). Cell in pro-anaphase (52 seconds). Early telophase, midbody has formed (54 seconds). See also Fig. 4A.
