# Supplementary material for: Programmed disassembly of a microtubule-based membrane protrusion network coordinates 3D epithelial morphogenesis in Drosophila
Source: EMBO J. 2024 Jan 23;43(4):5. doi: 10.1038/s44318-023-00025-w (PMC10897427; doi:10.1038/s44318-023-00025-w)
Supplement: Supplementary file 12 — Expanded View Figures [file 44318_2023_25_MOESM12_ESM.pdf]

Expanded View Figures

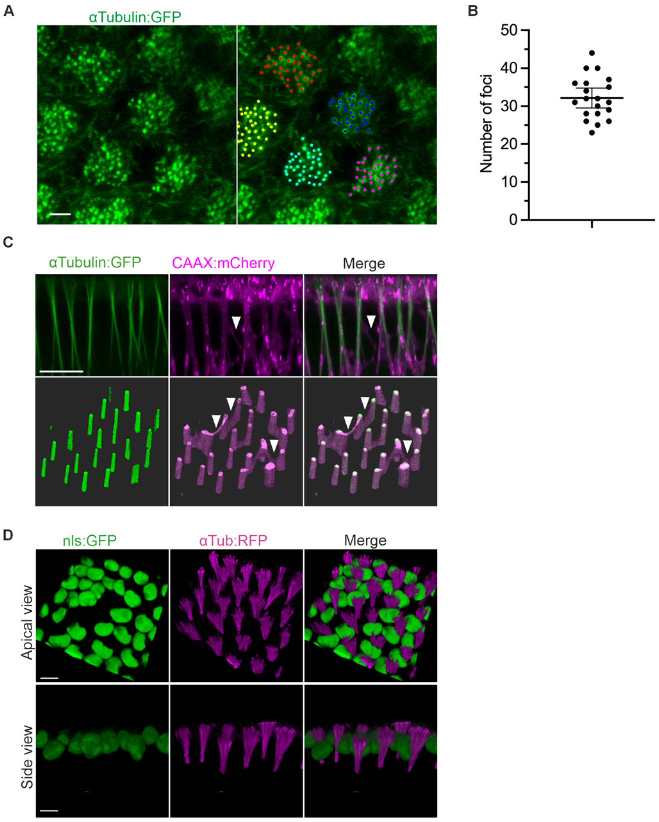

Figure EV1. Detailed structure of the IPAN.

(**A**) Apical view of MTs (left; green, αTubulin:GFP). Numbers of MT foci are counted (right; dots in various colors). (**B**) Number of foci of MTs in the apical compartment of each cell in (**A**). $n = 20$. Data are means ± 95% confidence intervals (CIs). (**C**) Both the vertical MT protrusions (green, αTubulin:GFP) and the horizontal MFs that connect them are enveloped in cell membrane (magenta, CAAX:mCherry). Upper panels provide an optical cross-sectional, and lower, an oblique view (apical towards the top of the page). White arrowheads point at lateral filopodia-like structures. (**D**) Cell nuclei are interspersed among the MT protrusions. Scale bars: 1 µm (**A**), 10 µm (upper panel in **C**), 5 µm (**D**).

 © The Author(s)

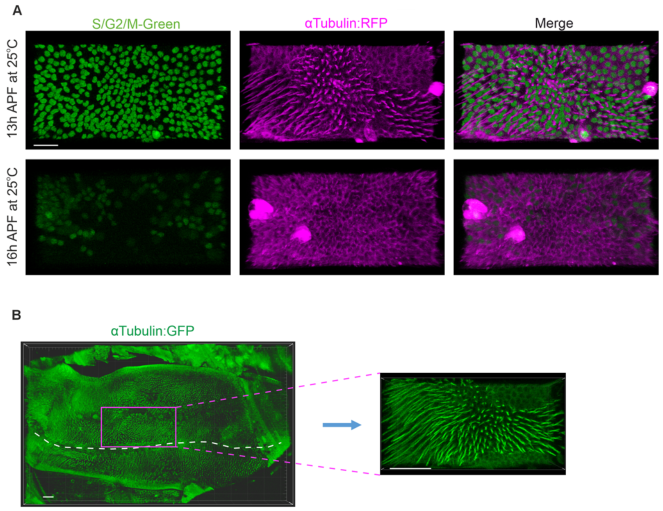

Figure EV2. Time-lapse imaging of cell cycle changes using S/G/M-green in pupal wing epithelial cells between 13 and 16 h APF.

(**A**) Our observations reveal that the majority of cells remain in the S/G2 phase during the early inflation stage (13 h APF at 25 °C). After 3 h (16 h APF at 25 °C), many cells enter mitosis. (**B**) Delimiting the region of interest (ROI) in which MT protrusion loss and mitoses are counted in dorsal and ventral epithelia. The ROI (magenta box) is adjacent to the trachea (white dashed line) close to the future hinge of the wing. Scale bar: 20 μm (**A**) and 30 μm (**B**).

© The Author(s)

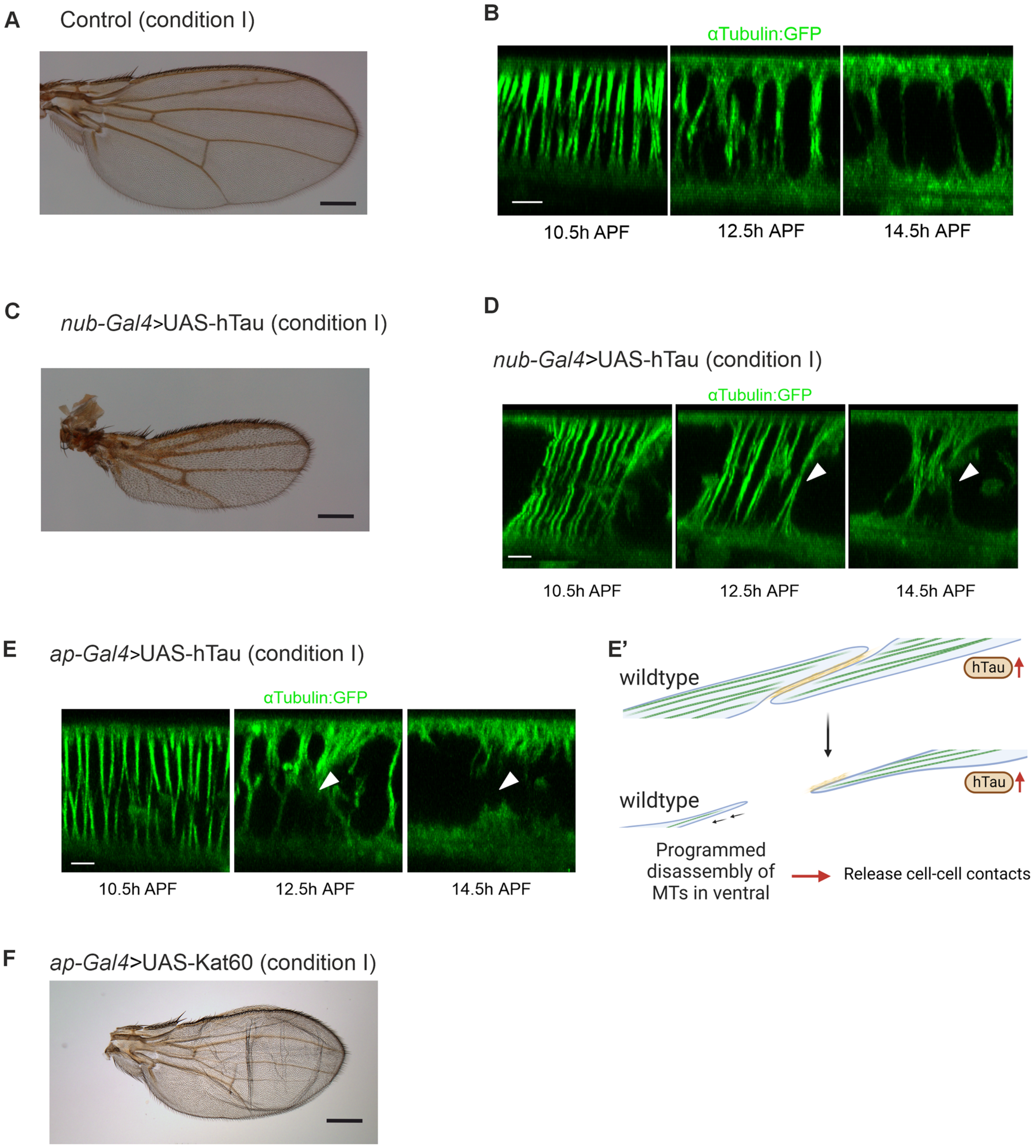

Figure EV3. Modulating MT stability affects wing morphogenesis.

(**A**) Adult wing in control condition I. (**B**) Lateral view of αTubulin:GFP of control at 10.5, 12.5, and 14.5 h APF. (**C**) Adult wing overexpressing hTau in both dorsal and ventral epithelium. (**D**) Lateral view of αTubulin:GFP during hTau overexpression in both dorsal and ventral layers (*nub-Gal4> hTau*) at 10.5, 12.5, and 14.5 h APF. (**E**) Lateral view of αTubulin:GFP during hTau overexpression in dorsal layers only (*ap-Gal4> hTau*) at 10.5, 12.5, and 14.5 h APF. Note that dorsal protrusions are thicker than ventral protrusions in hTau overexpression only in dorsal cells, which results in loss of cell–cell contacts (arrowheads). (**E'**) Schematics of the loss of cell–cell contacts in hTau oeverexpression only in dorsal cells. Disassembly of MT projections in ventral cells involves degeneration of the basal integrin-laminin complex, but not in dorsal cells, which is sufficient for leading to the loss of cell–cell contact. (**F**) Adult wing overexpressing Katanin60 in dorsal epithelium. Scale bars: 250 μm (**A**, **C**, **F**), 5 μm (**B**, **D**, **E**).

© The Author(s)

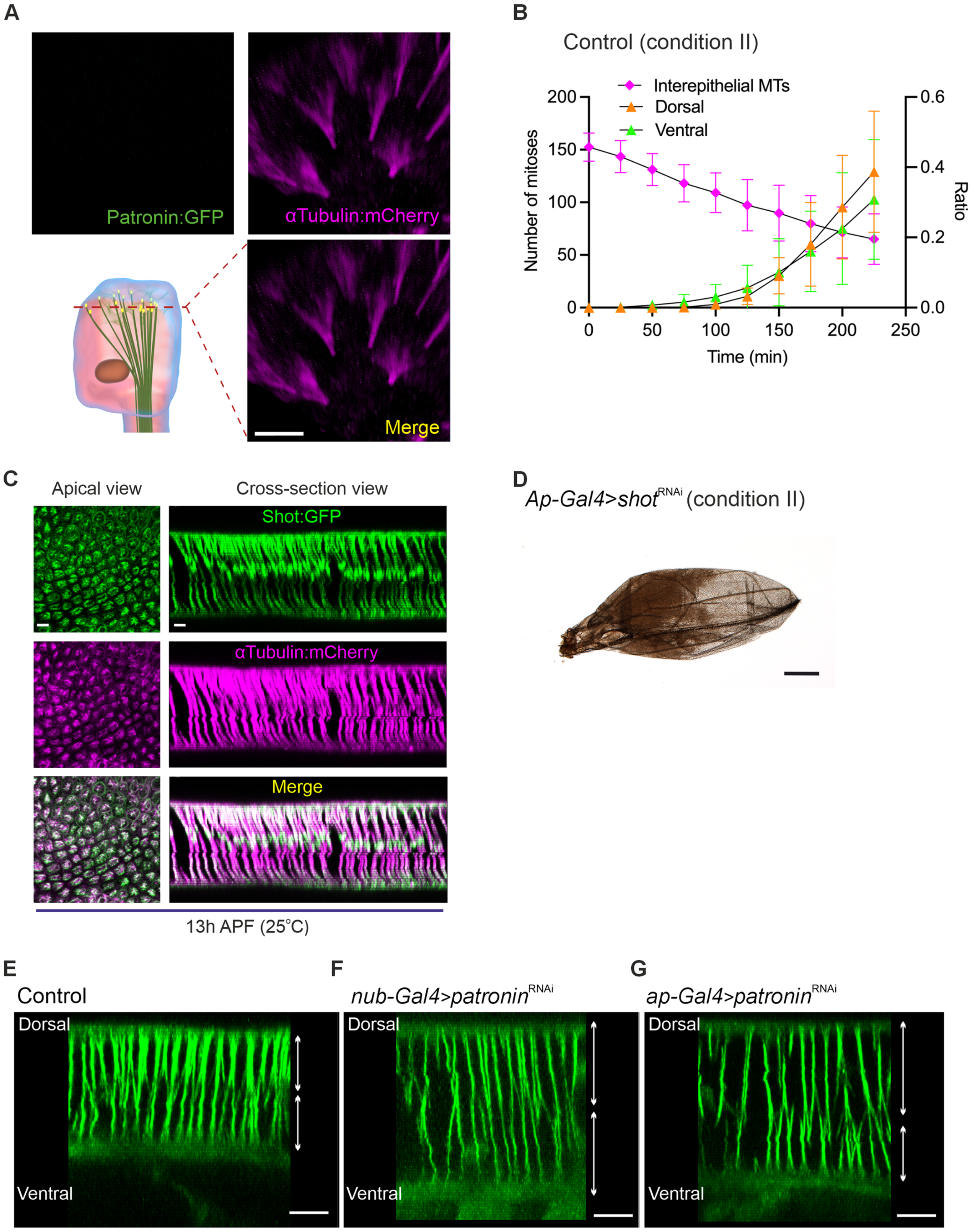
A
Patronin:GFP
αTubulin:mCherry
Merge
B
Control (condition II)
Interepithelial MTs
Dorsal
Ventral
Number of mitoses
Ratio
Time (min)
0
50
100
150
200
250
0.0
0.2
0.4
0.6
C
Apical view
Cross-section view
Shot:GFP
αTubulin:mCherry
Merge
13h APF (25°C)
D
Ap-Gal4>shotRNAi (condition II)
E
Control
F
nub-Gal4>patroninRNAi
G
ap-Gal4>patroninRNAi
Dorsal
Ventral

Figure EV4. MT-based protrusions are sustained by Patronin and Shot.

(**A**) Live imaging of αTubulin:mCherry (magenta) and Patronin:GFP (green) at 10.5 h APF (29 °C) during conditional RNAi of *patronin*, Note that Patronin:GFP signal diminishes after RNAi (compare to Fig. 7A). (**B**) Number of mitotic cells (dorsal: orange triangle, ventral: green triangle) in wing epithelium and ratio of interepithelial MT (magenta) at different time points in control pupal wings (condition II). Time 0 corresponds to 10.5 h APF. Data are from five individual replicates ($N = 5$). Data are means ± 95% CIs. (**C**) Shot:GFP localization in pupal wings. Live imaging of αTubulin:mCherry and Shot:GFP at 13 h APF (25 °C). Shot.GFP (top), αTubulin:mCherry (middle), merged image (bottom). (**D**) Adult wing of *shot* RNAi in dorsal epithelium. (**E–G**) Lateral view of αTubulin:GFP during control (**E**), conditional *patronin* RNAi in both dorsal and ventral layers (*nub-Gal4> patronin RNAi*, **F**) or only in dorsal layer (*ap-Gal4> patronin RNAi*, **G**). Note that protrusions are thinner and longer than control in *patronin*^{RNAi} in two layered epithelia (**E**, **F**), and ventral protrusions are thicker and shorter than dorsal protrusions during only dorsal *patronin*^{RNAi} (**G**). Scale bars: 5 μm (**A**, **C**), 250 μm (**D**), 15 μm (**E–G**).

 © The Author(s)

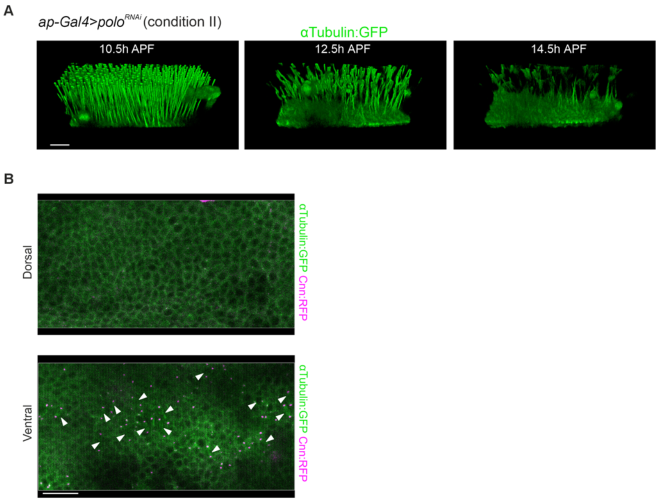

Figure EV5. Time lapse images of 3D view of MT protrusions visualized by αTubulin:GFP at 10.5, 12.5, and 14.5 h APF (29 °C) of the wing during conditional knockdown of *polo* (*ap > polo RNAi*).

(**A**) Apical surface of the dorsal epithelium is towards the top of the view. (**B**) Dorsal (top) and ventral (bottom) epithelial cells visualized by αTubulin:GFP (green) and Cnn:RFP (magenta) at 14.5 h APF. Note that mitotic cells are only observed in ventral epithelium (arrowheads). Scale bars: 20 µm (**A**, **B**).
